# Supplementary material for: Surveying the Proteome-Wide Landscape of Mitoxantrone and Examining Drug Sensitivity in BRCA1-Deficient Ovarian Cancer Using Quantitative Proteomics
Source: Proteomes. 2025 Nov 14;13(4):61. doi: 10.3390/proteomes13040061 (PMC12641731; doi:10.3390/proteomes13040061)
Supplement: Supplementary file 1 [file proteomes-13-00061-s001.zip › proteomes-3948485-supplementary.pdf]

Supplementary Information for:

Surveying the Proteome-Wide Landscape of Mitoxantrone and Examining Drug Sensitivity in BRCA1-Deficient Ovarian Cancer Using Quantitative Proteomics

Savanna Wallin[1], Sneha Pandithar[1], Sarbjit Singh[1], Siddhartha Kumar[1,2], Amarnath Natarajan[1,2], Gloria E. O. Borgstahl[1,*], Nicholas Woods[1,*]

[1] The Eppley Institute for Research in Cancer and Allied Diseases, Fred & Pamela Buffett Cancer Center, University of Nebraska Medical Center, Omaha, NE 68198, USA; savanna.wallin@unmc.edu (S.W.); spandithar@unmc.edu (S.P.); sarbjit.dhami@gmail.com (S.S.)

[2] Division of Pharmaceutical Sciences, College of Pharmacy, University of Cincinnati, Cincinnati, OH 45267, USA; kumar3sd@ucmail.uc.edu (S.K.); nataraah@ucmail.uc.edu (A.N.)

* Co-Correspondence: gborgstahl@unmc.edu (G.E.O.B); nicholas.woods@unmc.edu (N.W.)

Supplementary Information and Figures

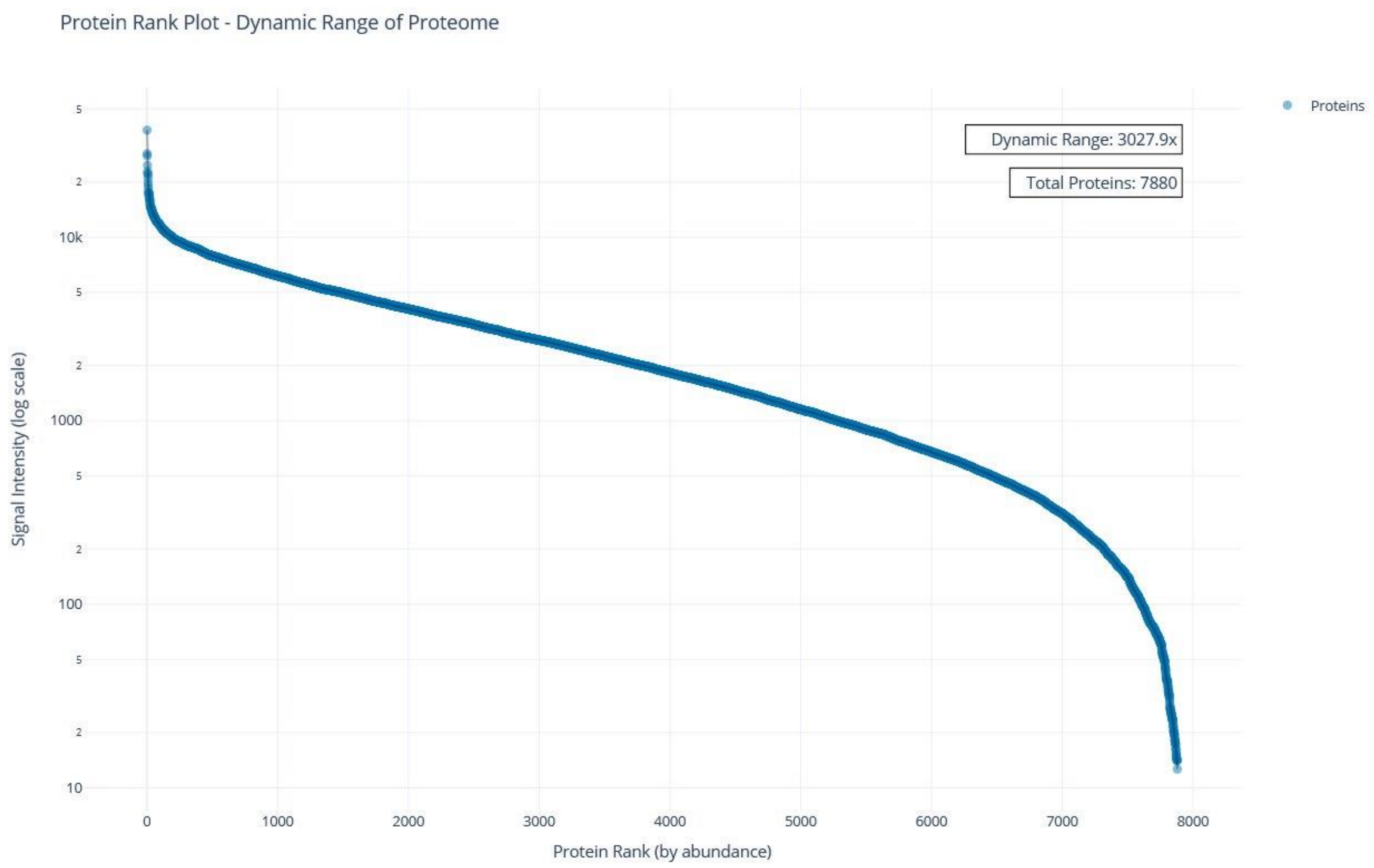

Supplementary Figure S1. Dynamic range of the shotgun TMT-labeled proteomics. A total of 7,880 proteins were identified in the TMT-labeled experiment comparing MX-treated and DMSO-treated BRCA1− and BRCA1+ UWB1.289 cells. The plot was generated using an in-house Python program.

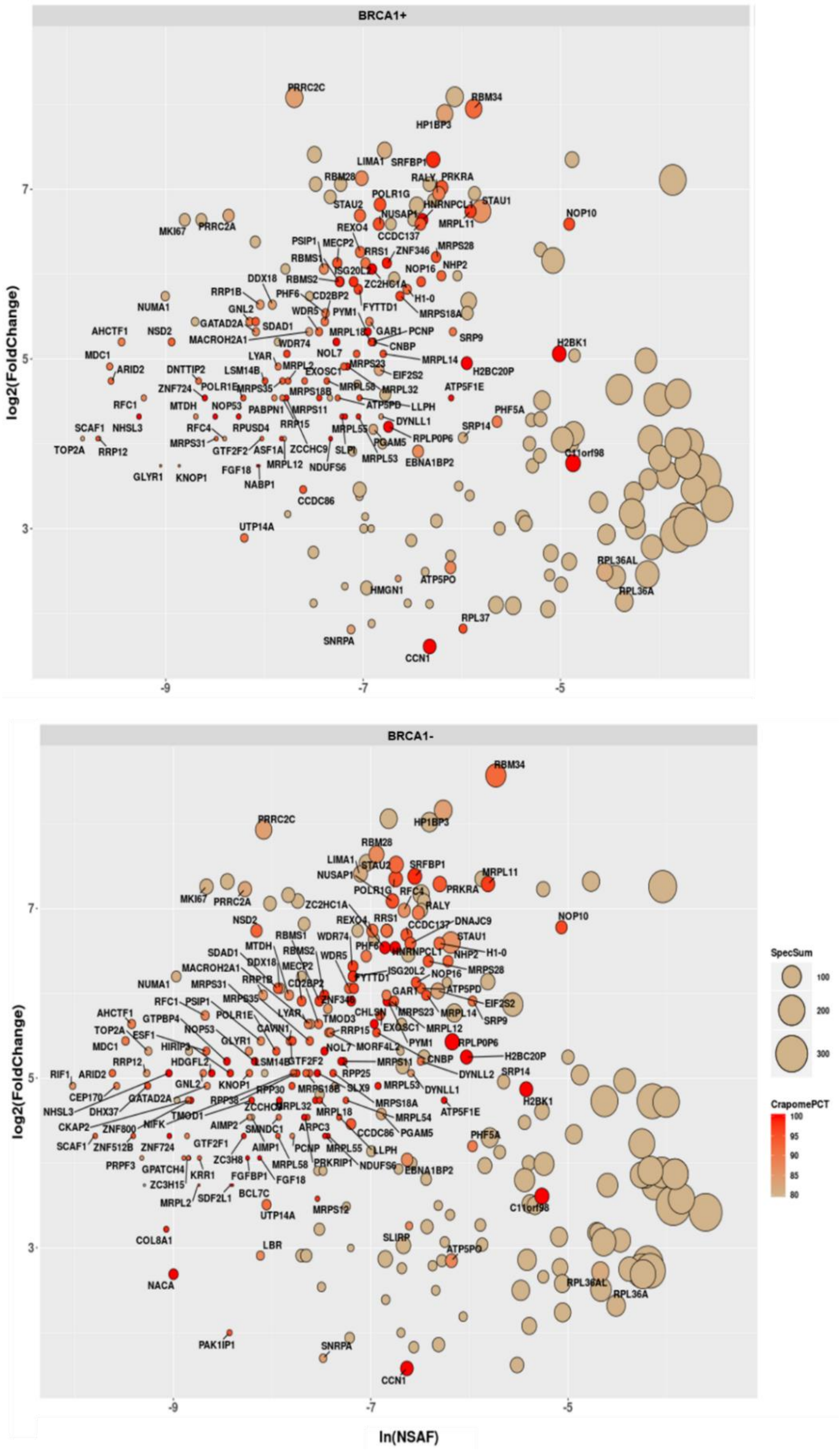

Supplementary Figure S2. A larger, higher-resolution image of Figure 4C. MXP-isolated proteins that met the SAINT score and CRAPomePCT thresholds are shown relative to their fold change and normalized spectral abundance factor (NSAF) compared to MXP-NC and DMSO controls. The overall abundance or spectral sum is represented by the size of the circle.

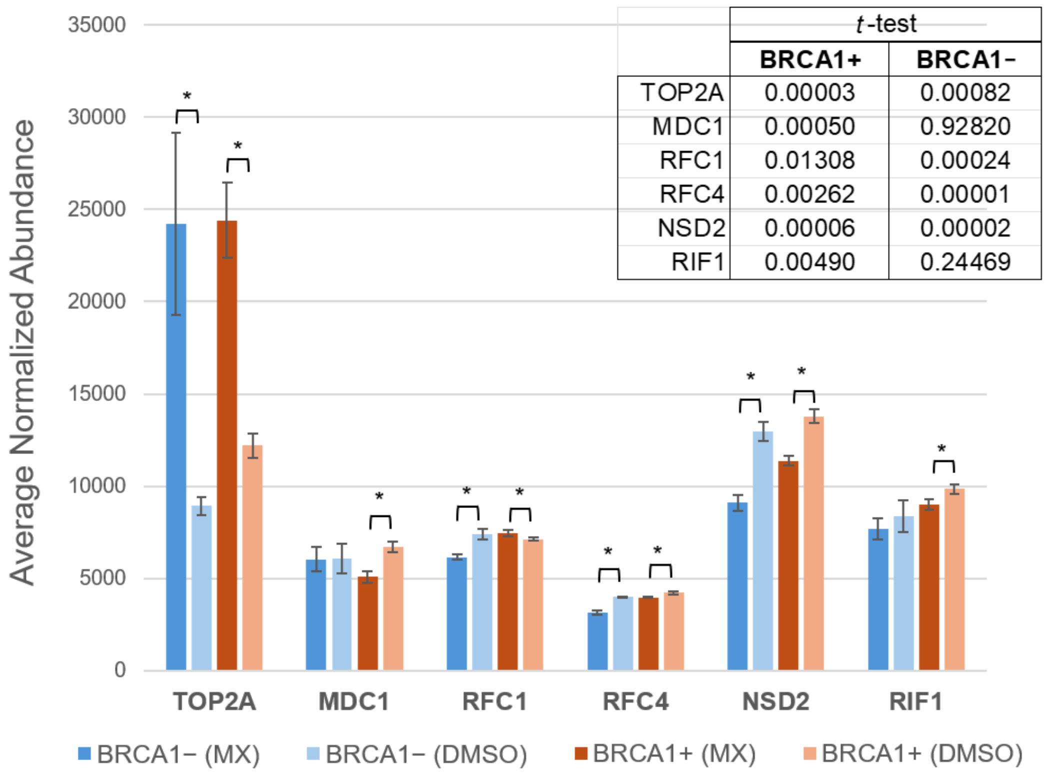

	t-test	
	**BRCA1+**	**BRCA1−**
TOP2A	0.00003	0.00082
MDC1	0.00050	0.92820
RFC1	0.01308	0.00024
RFC4	0.00262	0.00001
NSD2	0.00006	0.00002
RIF1	0.00490	0.24469

Supplemental Figure S3. Significant shifts in spectral abundance after MX-treatment. The average normalized spectra detected was compared in MX-treated and DMSO-treated samples in BRCA1− and BRCA1+ cells using an unpaired, two-tailed *t*-test with assuming equal variance ($p \leq 0.05$).

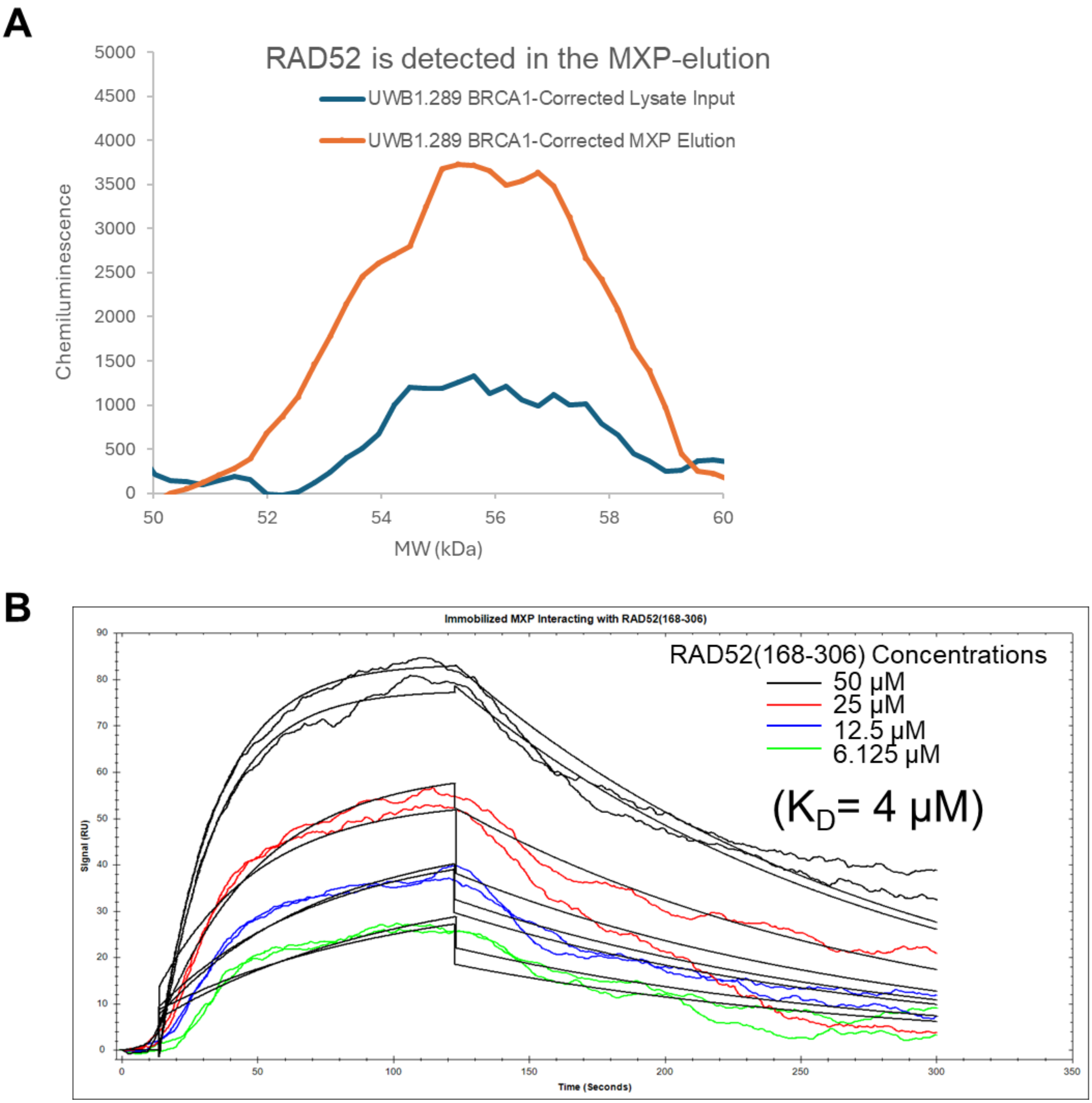

Supplemental Figure S4. Detection of RAD52 in the BRCA1-corrected (BRCA1+) UWB1.289 cell lysate and MXP-elution from BRCA1-corrected (BRCA1+) lysate. **(A)** RAD52, a weak binder of MX, was not detected in our mass spectrometry analysis. We suspected this was because it is a low-abundance protein. RAD52 was detected in the MXP-elution fraction with the peak at approximately 56 kDa using Peggy Sue Simple Western by ProteinSimple (Bio-Techne). The resulting electropherogram is pictured. We detected RAD52 in the lysate input, as well. **(B)** Surface plasmon resonance sensorgram depicting the association and dissociation of the RAD52 truncation RAD52(168-306) to and from immobilized MXP. The quantified dissociation constant (K_D) was approximately 4 μM.

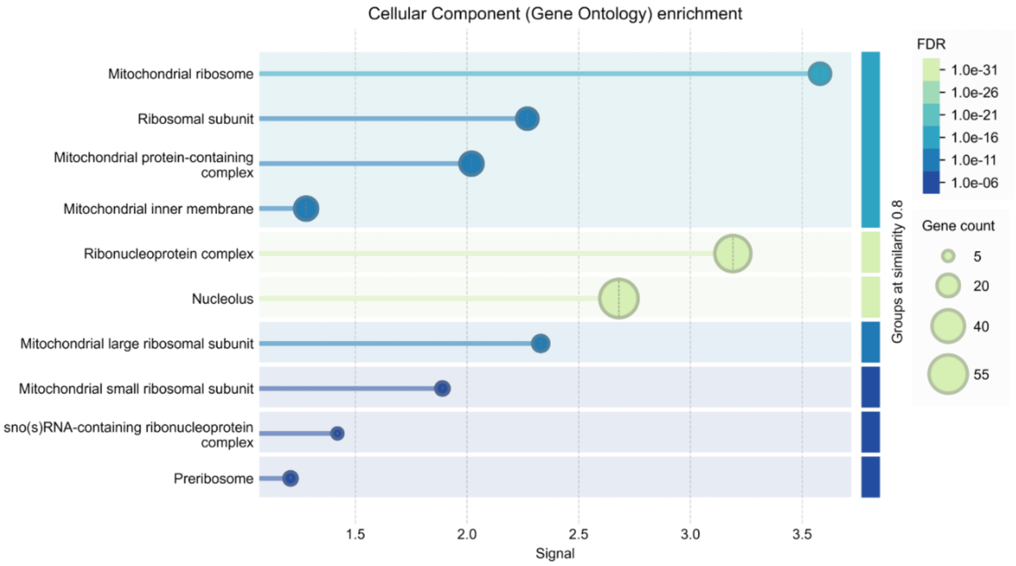

Supplemental Figure S5. Representative Gene Ontology: Cell Components gene enrichment of MXP-isolated proteins from parental and corrected cell extract. The graph was generated using STRING and terms were grouped with a 0.8 similarity.

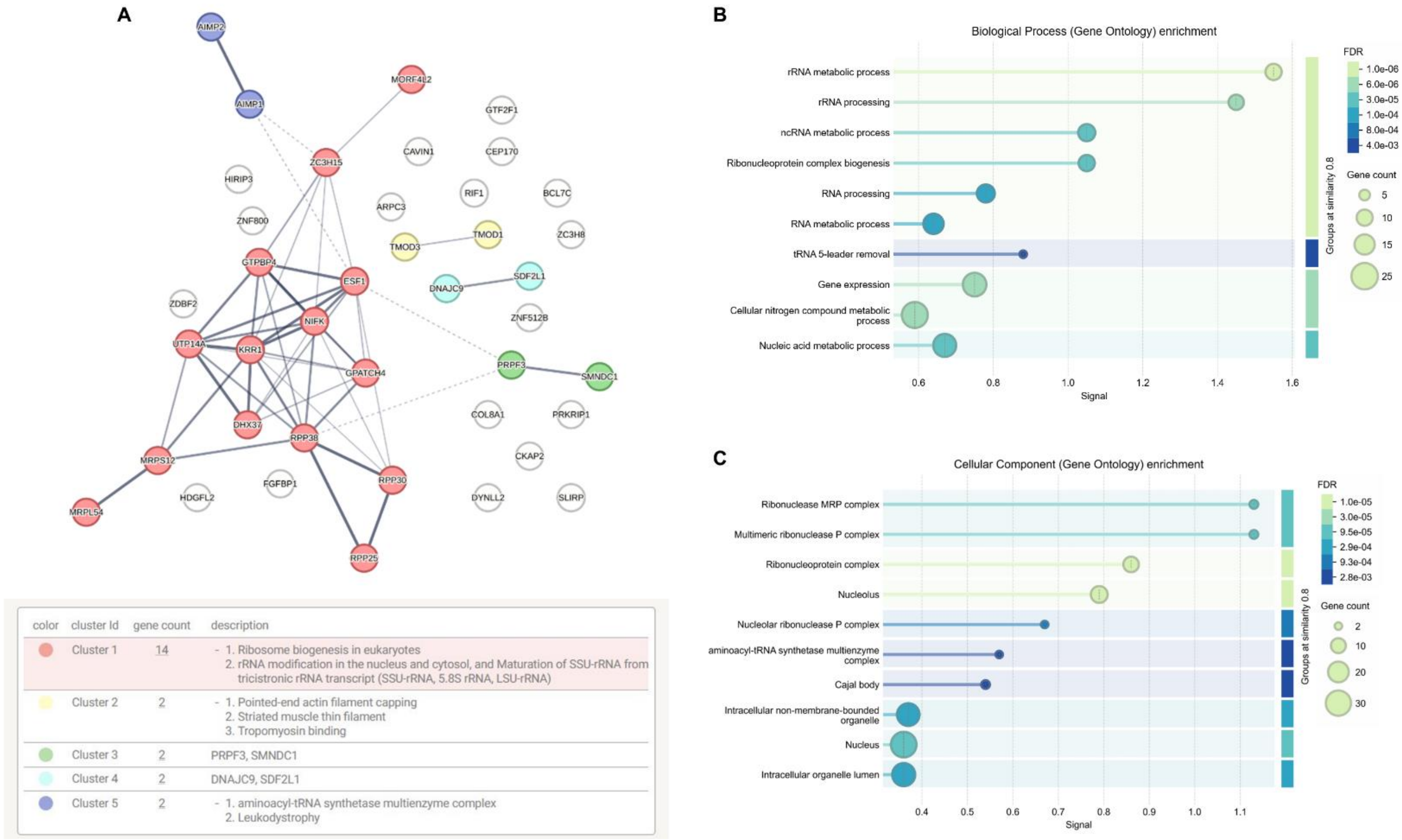

Supplemental Figure S6. MXP-isolated targets unique to parental UWB1.289 cells. **(A)** STRING analysis of the unique PMXP targets. The network was clustered using k-means clustering (5 clusters). The solid line indicates known interactions between nodes, and the intensity of the line increases with the confidence of the interaction. Dashed lines represent known interactions between clusters. Clusters are grouped functionally and physically, and STRING assigns a primary, and occasionally a secondary and tertiary description that best represents the cluster. The descriptions are based on gene ontology categories and are color-coded in the legend below the network. The number of genes categorized in each cluster is found in parentheses following the description. **(B)** STRING Gene Ontology (GO) biological process analysis (grouped with 0.8 similarity). **(C)** STRING GO analysis of cellular components (grouped with 0.8 similarity).

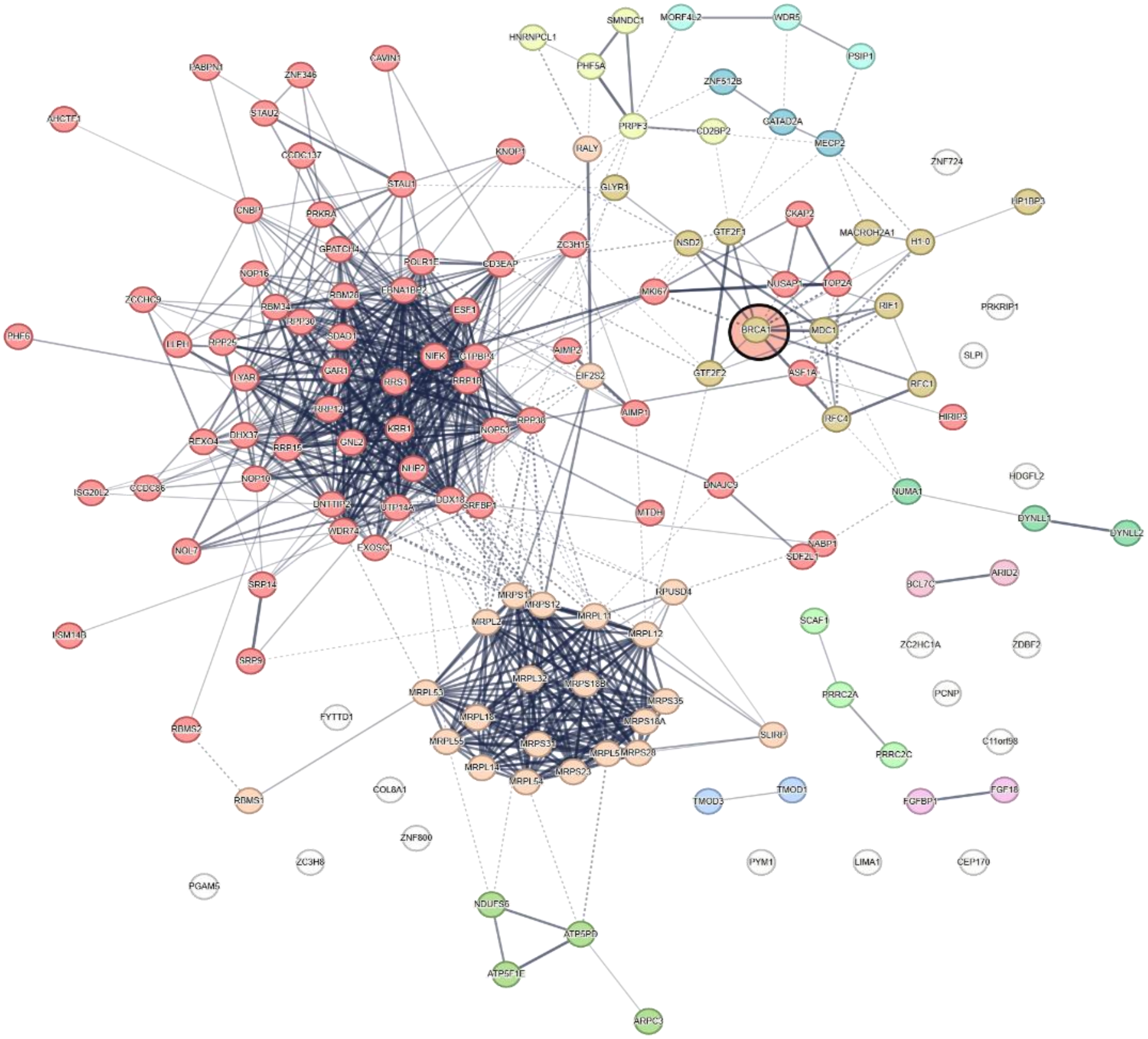

color	cluster Id	gene count	description
	Cluster 1	66	rRNA metabolism
	Cluster 2	23	Mitochondrial translation
	Cluster 3	12	- 1. Nonhomologous End-Joining (NHEJ)<br>2. Nucleosome binding<br>3. Breast cancer carboxy-terminal domain
	Cluster 4	5	Spliceosomal tri-snRNP complex assembly
	Cluster 5	4	+ Formation of ATP by chemiosmotic coupling
	Cluster 6	3	PRRC2A, PRRC2C, SCAF1
	Cluster 7	3	Dynein light chain type 1
	Cluster 8	3	H4 histone acetyltransferase complex
	Cluster 9	3	Sin3 complex, and NuRD complex
	Cluster 10	2	- 1. Pointed-end actin filament capping<br>2. Striated muscle thin filament<br>3. Tropomyosin binding
	Cluster 11	2	FGFR2 ligand binding and activation
	Cluster 12	2	SWI/SNF complex

Supplemental Figure S7. STRING analysis of MXP-targets identified from parental and BRCA1-corrected UWB1.289 cells with BRCA1. This network demonstrates the functional relationship some MXP-targets have with BRCA1, but it also shows distant and unrelated targets that potentially contribute to cytotoxic activity.
